# Preservation artifacts in zebrafish X-ray fluorescence imaging

**DOI:** 10.1093/mtomcs/mfag026

**Published:** 2026-08-24

**Authors:** Ashley K James, Monica Weng, Natalia V Dolgova, Kelly L Summers, Graham N George, Ingrid J Pickering

**Affiliations:** Toxicology Centre, University of Saskatchewan, 44 Campus Drive, Saskatoon, Saskatchewan S7N 5B3, Canada; Molecular and Environmental Sciences Group, Department of Geological Sciences, University of Saskatchewan, 114 Science Place, Saskatoon, Saskatchewan S7N 5E2, Canada; Molecular and Environmental Sciences Group, Department of Geological Sciences, University of Saskatchewan, 114 Science Place, Saskatoon, Saskatchewan S7N 5E2, Canada; Molecular and Environmental Sciences Group, Department of Geological Sciences, University of Saskatchewan, 114 Science Place, Saskatoon, Saskatchewan S7N 5E2, Canada; Molecular and Environmental Sciences Group, Department of Geological Sciences, University of Saskatchewan, 114 Science Place, Saskatoon, Saskatchewan S7N 5E2, Canada; Toxicology Centre, University of Saskatchewan, 44 Campus Drive, Saskatoon, Saskatchewan S7N 5B3, Canada; Molecular and Environmental Sciences Group, Department of Geological Sciences, University of Saskatchewan, 114 Science Place, Saskatoon, Saskatchewan S7N 5E2, Canada; Department of Chemistry, University of Saskatchewan, Saskatoon, SK S7N 5C9, Canada; Toxicology Centre, University of Saskatchewan, 44 Campus Drive, Saskatoon, Saskatchewan S7N 5B3, Canada; Molecular and Environmental Sciences Group, Department of Geological Sciences, University of Saskatchewan, 114 Science Place, Saskatoon, Saskatchewan S7N 5E2, Canada; Department of Chemistry, University of Saskatchewan, Saskatoon, SK S7N 5C9, Canada

## Abstract

Preservation and embedding procedures can substantially influence elemental distributions measured by X-ray fluorescence imaging (XFI), particularly for mobile endogenous elements. While effects of formalin fixation have been widely studied, comparatively little is known regarding methacrylate embedding. Here, larval zebrafish (*Danio rerio*) exposed to methylmercury—l-cysteinate (MeHg-l-Cys) were preserved either by JB-4 methacrylate embedding or using cryosectioning/air-drying, called herein cryopreservation, then were sectioned and analyzed using quantitative XFI. Mercury distributions were largely unaffected by preservation method, whereas endogenous elements including potassium, calcium, copper, sulfur, selenium, and zinc showed varying degrees of depletion or redistribution following methacrylate embedding, with potassium showing particularly strong depletion. Despite these changes, methacrylate embedding provided improved anatomical preservation relative to cryopreservation. These results demonstrate that methacrylate embedding is suitable for studies focused primarily on mercury localization, while cryopreservation remains highly preferable when accurate preservation of endogenous elemental distributions is required.

## Graphical Abstract

**Graphical Abstract:**
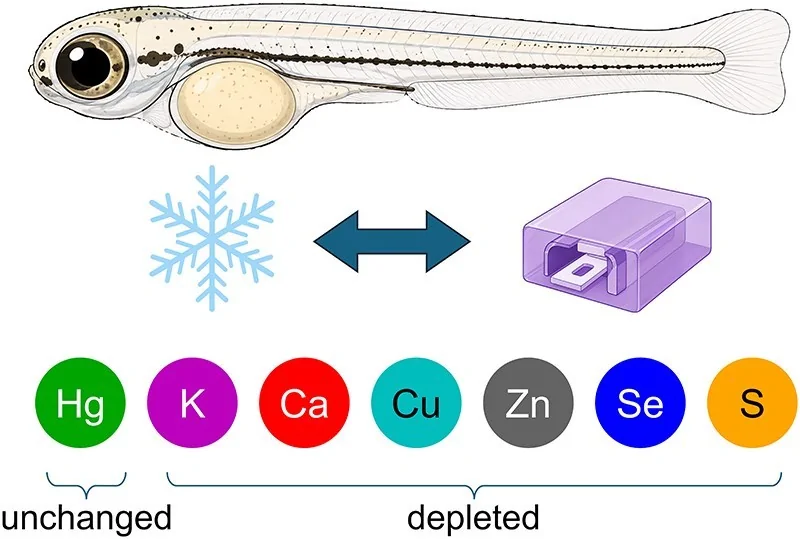
Cryopreservation maintains endogenous elemental distributions, while methacrylate embedding improves anatomical preservation without altering mercury distributions.

## Introduction

Larval stage zebrafish (*Danio rerio*) are an important vertebrate model for studies of development, toxicology, and metallomics [1,2]. The widespread use of larval zebrafish reflects the high fecundity of the adult fish together with the small size, near optical transparency, rapid development, and strong conservation of vertebrate biochemical pathways during larval development [3]. Approximately 70% of human genes have at least one zebrafish orthologue, with over 80% of human disease-associated genes being represented in the zebrafish genome [3]. These characteristics have led to widespread adoption of larval stage zebrafish as the de-facto standard for early-stage pharmaceutical screening and first-pass vertebrate toxicological assessment [2].

**Figure 1:**
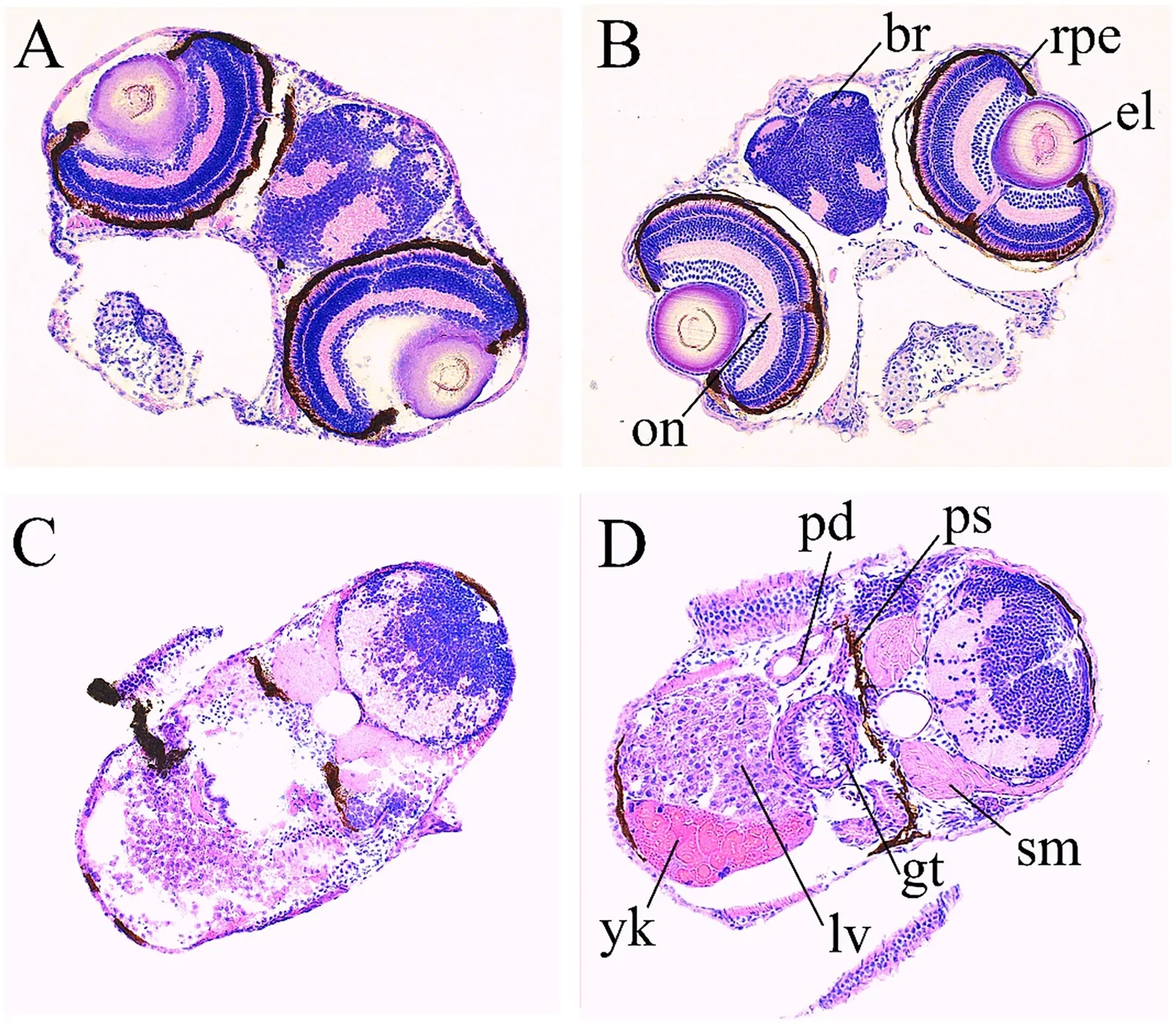
Optical micrographs of 10 µm head (A, B) and liver sections of 5 day post-fertilization larval zebrafish using cryo-sectioning (A, C) or methacrylate embedding (B, D). A representative image of a head section (B) denotes various important anatomical features including the eye lens (el), brain (br), optical nerve (on), retinal pigmented epithelium (rpe) while the liver section (D) shows liver (lv), yolk sac (yk), gut tube (gt), pronephric duct (pd), somatic muscle (sm), and pigment spot (ps).

X-ray fluorescence imaging (XFI) [4,5] has emerged as a powerful approach for mapping elemental distributions in intact zebrafish tissues and whole larvae in studies of metal uptake, localization, and toxicity [6–17]. XFI measurements are frequently performed on sectioned specimens, where preservation and embedding procedures may substantially influence measured elemental concentrations and localizations, particularly for mobile ions and diffusible intracellular species [4,5,18–21]. Cryopreservation is generally regarded as a method that will faithfully preserve endogenous elemental distributions. Methacrylate embedding provides excellent anatomical preservation and remains a standard in zebrafish studies, especially in histological work [22]. Figure 1 shows typical histological micrographs of hematoxylin/eosin-stained sections of both head and trunk sections which have been either cryopreserved (Fig. 1A and C) or methacrylate embedded (Fig. 1B and D). The preservation of tissue anatomy is significantly better for the section derived from methacrylate embedded larva (Fig. 1B and D).

Here, we compare cryopreservation and methacrylate embedding for XFI analysis of larval zebrafish exposed to methylmercury—l-cysteinate (MeHg-l-Cys). Using quantitative elemental imaging, we examine the extent to which preservation method influences both elemental localization and measured concentrations for mercury and endogenous biological elements across multiple tissue types.

## Experimental

### Zebrafish husbandry and MeHg-l-Cys exposure

Zebrafish (wildtype, Tübingen strain) were raised and maintained in the Lab Animal Services Unit (LASU) at the University of Saskatchewan under protocols approved by the University of Saskatchewan Animal Ethics Board. Embryos, collected following mating, were rinsed with system water and maintained in embryo medium in Petri dishes (≤50 embryos/dish) at 28°C, with daily medium replacement and removal of debris, chorions, and dead embryos. At 3 days post-fertilization (dpf), after hatching was complete, larvae were transferred to treatment dishes containing 20 larvae per dish with a minimum of five dishes per treatment group. MeHg-l-Cys stock solutions were prepared from methylmercury hydroxide (Strem Chemicals Inc., Newburyport, MA), l-cysteine (Millipore Sigma, Oakville, ON), and deionized water as previously described [6,7]. Treatment groups consisted of control larvae (embryo medium only) and larvae exposed to 0.2 µM MeHg-l-Cys. Exposure solutions were renewed every 24 h, with mortality and deformities recorded daily. Exposures continued until 5 dpf, after which surviving larvae were euthanized using 0.2% tricaine.

### Sample preparation

Two preservation methods were employed. For cryopreservation, euthanized larvae were embedded in Tissue-Tek optimal cutting temperature media (Skaura Finetek, Torrance CA, USA) and then were rapidly frozen in liquid nitrogen-cooled isopentane [23,24]. Samples were then cryosectioned, air-dried and transported at room temperature to the Advanced Photon Source for XFI measurements. In this work, cryopreserved refers to cryosectioned and air-dried specimens; we did not use frozen-hydrated or cryogenic XFI. For methacrylate preservation, larvae were fixed in 4% paraformaldehyde, dehydrated through graded ethanol washes, infiltrated with JB-4 solutions (Polysciences, Warrington, PA, USA), with these processing steps completed over several hours during the same day. Samples were then polymerized overnight at 4°C. Samples from both methods were sectioned at 10 µm. Sections for X-ray fluorescence imaging (XFI) were mounted on Thermanox coverslips, while adjacent sections for histology and optical microscopy were mounted on Fisherbrand Superfrost Plus slides.

### X-ray fluorescence imaging

XFI maps were collected at beamline 20-ID-B of the Advanced Photon Source (APS, Argonne, IL; subsequently relocated to Sector 25 during the APS upgrade) operating in top-up mode at 102 mA. A Si(111) double-crystal monochromator and Rh-coated Kirkpatrick—Baez mirrors provided a ∼5 × 5 µm^2^ focused beam at 13 450 eV. Samples mounted on 6.3 µm polypropylene film were raster scanned with 5 × 5 µm^2^ pixels and a dwell time of 0.6 s/pixel. X-ray fluorescence was detected at 90° to the incident beam using a collimated 4-element silicon drift detector array (Hitachi High-Technologies Science America Inc., Northridge, CA, USA). XFI maps were processed and visualized with the MicroAnalysis Toolkit (SMAK) software [25], which employs X-ray fluorescence spectral fitting routines from PyMCA [26]. Anatomical regions within XFI maps used for quantification were defined by using the mask tool contained within the SMAK software, excluding any voids in which tissue was not present. This process was assisted by examining the transmitted X-ray beam and examining the accompanying histological sections.

### Statistical considerations

Areal densities derived from XFI measurements arise from photon-counting processes, which can only hold positive values; a Poisson distribution will correctly reflect the probability distribution function of photon-counting processes [27,28]. However, XFI elemental maps are derived from spatially heterogeneous biological systems and additional processing steps including background subtraction, normalization, and spectral fitting. Consequently, the resulting distributions of elemental areal densities are continuous quantities that are strictly non-negative. At low signal levels, symmetric Gaussian descriptions can therefore become problematic when their variance is sufficiently large to assign appreciable probability to negative values. Positive-valued distributions such as the gamma distribution [29,30] provide a useful illustration of this behaviour. The continuous gamma probability density function $f( {x;k,\theta } )$ is given by eq. 1


(1) \begin{eqnarray*}
f\left( {x;k,\theta } \right) = \frac{{{{x}^{k - 1}}{{e}^{ - x/\theta }}}}{{{\mathrm{\Gamma }}\left( k \right){{\theta }^k}}}.
\end{eqnarray*}

**Figure 2:**
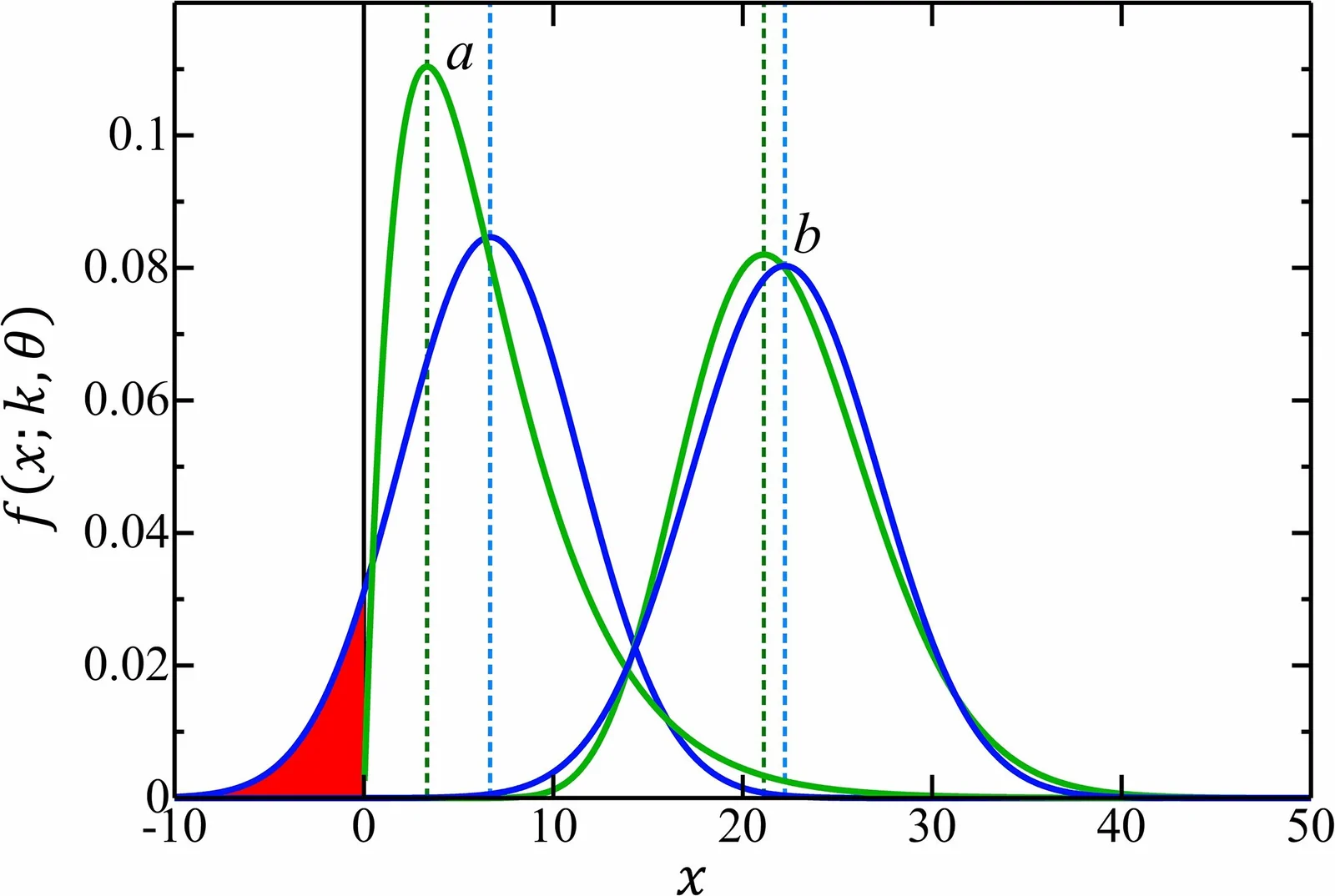
Gamma probability distribution functions (green solid lines) representative of low (*a*) and high (*b*) count-rate measurements, together with equivalent normal (Gaussian) distributions (blue solid lines). The broken blue lines show the means for the two distributions, and the broken green lines show the mode for the gamma distribution; the modes for the Gaussians are equivalent to the means. The red filled area under the normal distribution shows the physically unrealistic region. Curves *a* were calculated with *k*=2 and *θ *=3.33 and *b* with *k*=20 and *θ *=1.11.

Here *x* is the measured areal density, *k* is a dimensionless shape parameter, *θ * is the scale parameter and ${\mathrm{\Gamma }}( k )$ is given by ${\mathrm{\Gamma }}( k ) = \mathop \smallint \limits_0^\infty {{t}^{k - 1}}{{e}^{ - t}}dt$, where *t* is an integration variable. The mean is given by $\mu = k\theta $, the variance by ${{\sigma }^2} = k{{\theta }^2}$, and the mode by $( {k - 1} )\theta $. Unlike the normal distribution, the gamma distribution is defined only for positive values and naturally accommodates right-skewed heteroscedastic behavior that is expected at low concentrations. It progressively approaches a Gaussian normal distribution as the shape parameter *k* becomes large [30]. Figure 2 shows example plots of $f( {x;k,\theta } )$, representative of low and high concentration conditions, showing that Gaussian statistics are expected to be a reasonable approximation for high concentrations, but not for low.

Accordingly, positive-valued statistical descriptions, such as the gamma distribution, may provide a more appropriate representation of uncertainties and concentration distributions in low-signal XFI measurements relative to conventional Gaussian approximations. Inherently non-negative XFI data must show a heteroscedastic variance that will scale with signal intensity. Consequently, symmetric homoscedastic Gaussian models become problematic, particularly near detection limits or for strongly depleted elements. Moreover, statistical tests based on normal-distribution assumptions (e.g. two sample *t*-tests) may underestimate true *P*-values by treating high-intensity observations as rarer than they are under the correct underlying distribution, particularly for small sample sizes *n*. If the number of samples *n* is not limiting, then a positive value distribution, such as a gamma model, can be used to describe the statistics. Unfortunately, because of the scarcity of beamtime the norm for XFI is *n*= 2 to 5, in which case a gamma fit (for example) will be underdetermined. In our case cryopreserved MeHg-l-Cys treatment groups had *n* = 4, while methacrylate-preserved groups had *n* = 3 for head sections and *n* = 2 for trunk sections.

Error bars based on standard deviations are typically presented because they remain a widely used and visually familiar means of representing experimental variability in quantitative biological literature, although their interpretation is less rigorous for asymmetric distributions such as those expected for XFI-derived areal densities. To avoid zero-crossing, here we use error bars spanning *2σ *, where *σ * is the standard deviation, in logarithmic space, as described below.

## Results and discussion

Before considering preservation-specific effects, we will briefly consider other sources of variability. Small differences in section depth can alter the relative volumes of organs present within a section and thus influence XFI quantitation, especially with whole-section regions. For example, shallow head sections contain proportionally less eye lens and more brain tissue than deeper sections. Additional variability may arise from biological differences between larvae, elemental heterogeneity within tissues, and minor differences in section thickness. Although section thickness variations are expected to be small here, consistent sectioning is important and is generally easier to achieve with methacrylate-embedded tissues than with cryopreserved samples, which tend to be more mechanically fragile. For a review of microtomy using frozen and fixed tissues, see Spencer et al. [24]. Regions of interest were defined anatomically. Head sections were divided into brain, eye lens, and whole-section regions, while trunk sections were divided into pronephric duct (kidney), liver, and whole-section regions.

**Figure 3:**
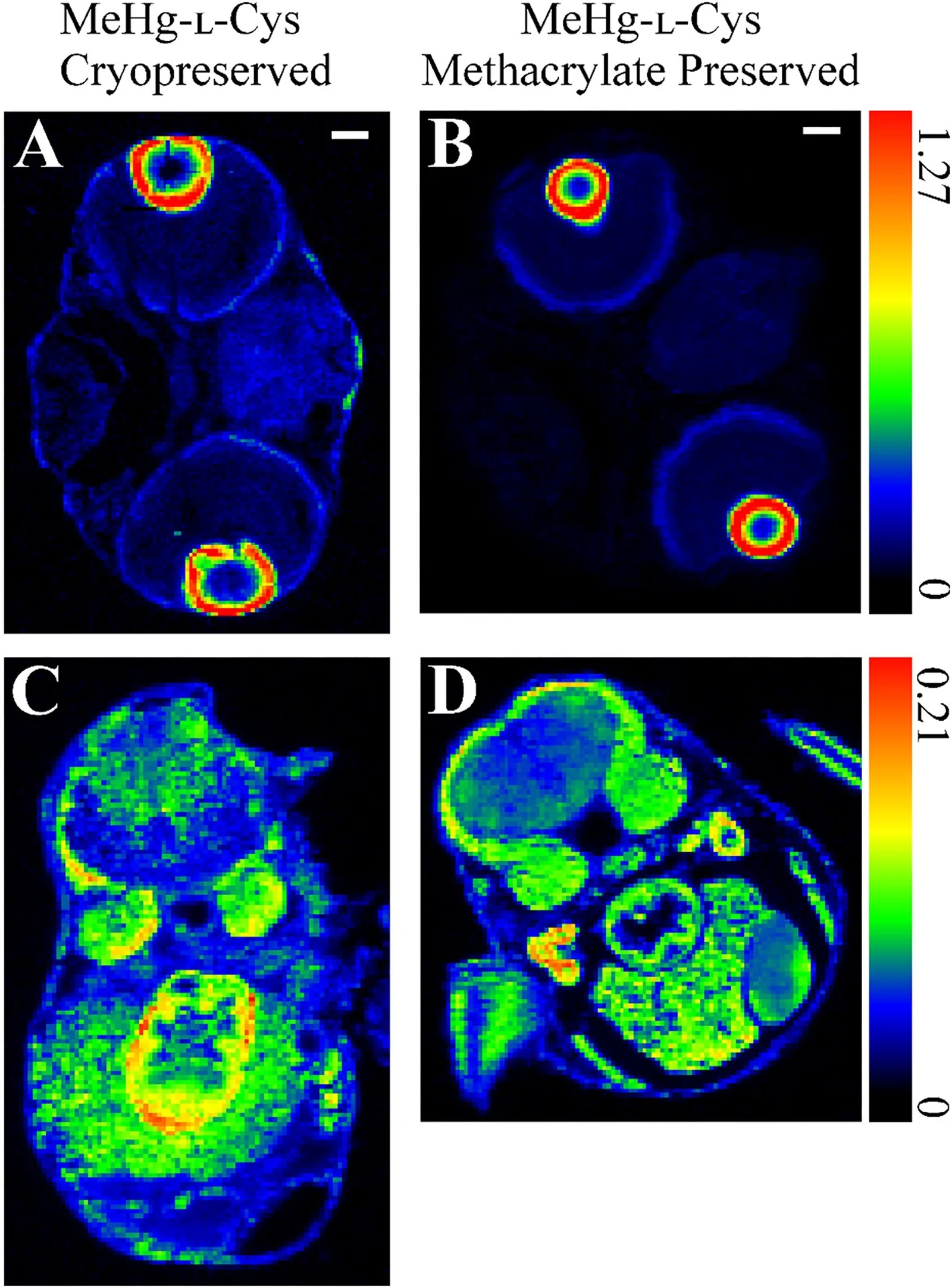
Hg XFI of head (A, B) and trunk (C, D) sections of 5 dpf zebrafish larvae following 48 h exposure to 0.2 µM MeHg-l-Cys, comparing cryopreserved (A, C) and methacrylate preserved (B, D) samples. Methacrylate-preserved images were scaled to the maximum Hg concentration observed in cryopreserved sections. Colour bars indicate Hg areal density (µg/cm^2^). Scale bar = 50 µm. Images are presented at the same scale.

**Figure 4:**
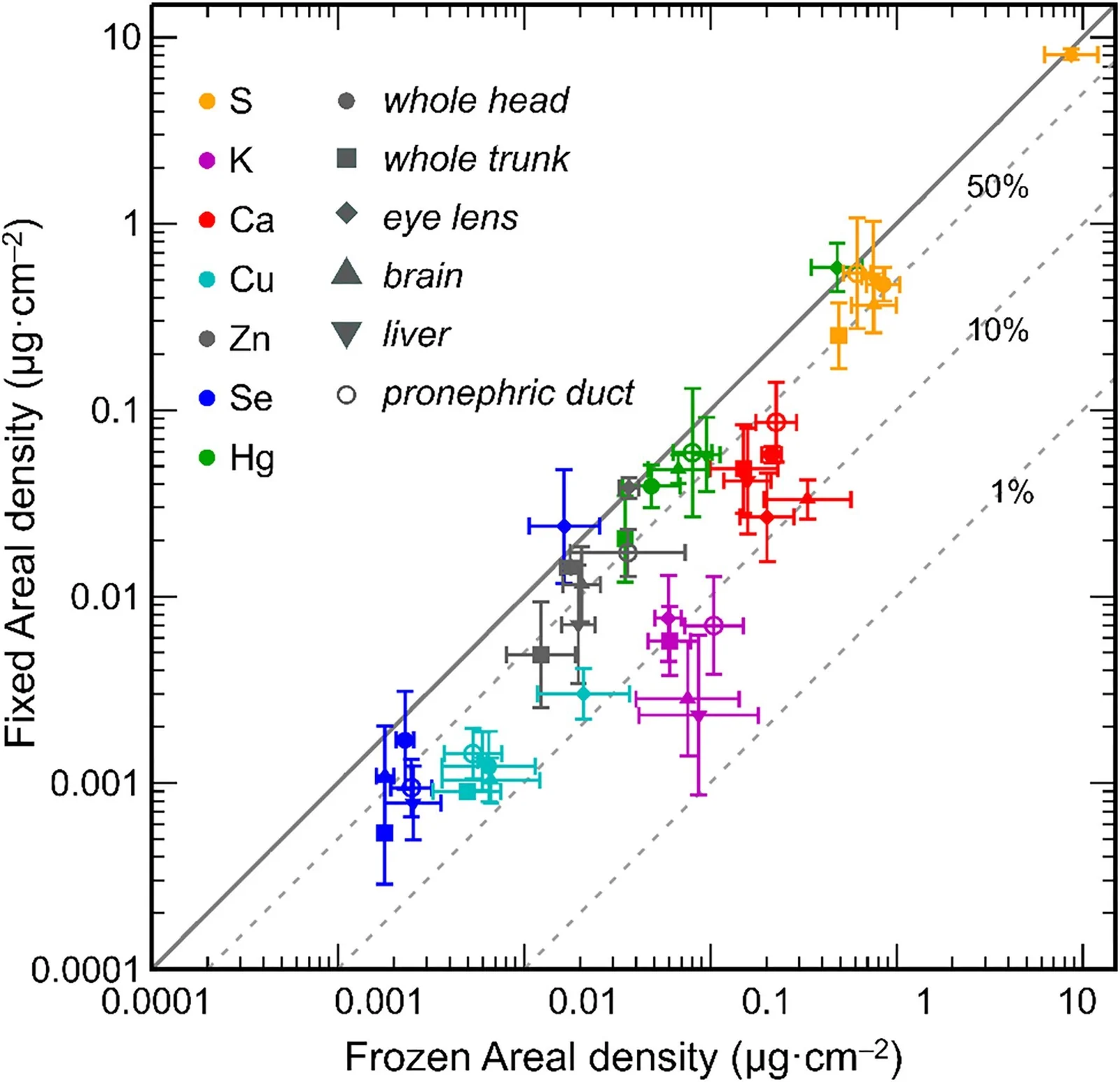
Plot of mean areal densities for seven different elements, comparing cryopreserved (frozen) versus methacrylate preserved (fixed). Mean areal densities in µg/cm^2^ (log_10_ scale) are plotted from equivalent representative organs with colors denoting individual elements, while points correspond to eye lens, brain, liver, kidney, and whole head and trunk regions. The solid diagonal indicates equal values for the two preservation methods, while dashed lines indicate fixed values corresponding to 50%, 10%, and 1% of frozen values. Each point represents the mean elemental concentration for the indicated anatomical region; error bars represent the standard deviation among the biological replicates.

Representative Hg Lα XFI data for head and trunk sections of 5 dpf larvae exposed to 0.2 µM MeHg-l-Cys for 48 h are shown in Fig. 3. Although the improved anatomical preservation afforded by methacrylate embedding is evident in Fig. 1, these differences are not apparent at the tissue-level spatial resolution of the XFI measurements shown in Fig. 3. At this resolution, both preparation methods preserve the major anatomical features necessary for comparison of elemental distributions.A plot of selected elements is shown in Fig. 4, as a log_10_-log_10_ plot. Conventional $\bar{x} \pm \sigma $ intervals extended below zero for some low-concentration measurements and hence cannot be displayed on a logarithmic scale. Instead, the standard deviations were represented empirically by positive intervals that are symmetric in log_10_ space. For each mean $\bar{x}$ and standard deviation *σ *, a logarithmic half-width was defined by $d = {\mathrm{asinh}}( {\sigma /\bar{x}} )/\ln 10$. The inverse hyperbolic sine provides a smooth transition from linear behaviour at small relative standard deviations to logarithmic behaviour at larger values, ensuring positive intervals with respective lower and upper limits of $\bar{x}{{10}^{ - d}}$ and $\bar{x}{{10}^{ + d}}$ with a linear span of *2σ *, and reduce to conventional $\bar{x} \pm \sigma $ limits when $\sigma /\bar{x}$ is small. They are used as a graphical representation of variability and do not constitute conventional confidence intervals.

Iron was not included in Fig. 4 because it showed little regional variation within the larvae, although it was consistently lower in the fixed samples than in the cryopreserved specimens. In contrast to the present study, Gellein et al. [33] reported little change in Fe following formalin fixation, whereas Cu, Zn, and Hg increased progressively with prolonged formalin storage. The differing observations may reflect the substantially shorter fixation and processing times used here (hours rather than the weeks to years examined by Gellein et al. [33]), differences in analytical scale (synchrotron XFI of thin sections versus bulk ICP-MS), and differences in fixation chemistry, as Gellein et al. used commercial formalin containing methanol whereas paraformaldehyde was used in the present study.

For mercury, the distributions show significant similarity between cryopreserved and methacrylate preserved sections. Established target organs for MeHg-l-Cys include the periphery of the eye lens and the brain, as well as the liver and kidneys. Cryopreserved samples showed slightly higher average levels in the brain and the whole section, although these differences may not be significant. Likewise, the average mercury levels of the eye lenses for the methacrylate were slightly higher, but this also may not be significant. Slightly higher mercury levels were observed for cryopreserved trunk sections, specifically in the liver and the pro-nephric duct (kidneys) as well as the entire trunk sections encompassing all tissues present, but once again these are subtle differences that may not be significant. In agreement with this, the mercury points in Fig. 4 are all clustered near, albeit slightly below, the equivalency line and the error bars for all mercury points are very close to the equivalency line.

Likewise, selenium and sulfur levels are only subtly affected by the preservation method. Sulfur shows relatively high levels with all fixed values falling between 50% and 100% of those the frozen in Fig. 4. Selenium shows lower levels and correspondingly higher error bars with some points falling to below 50%. Zinc levels also change slightly with preservation method. In previous work we have observed zinc-colocalizing with pigment spots [12], thought to be predominantly eumelanin polymers contained within melanophores [31]. While pigment spots were not specifically included in our organ-specific quantification, for trunk sections, zinc was seen in both preservation methods to accumulate highly in the dorsal, midline, and ventral pigment spots, and we empirically observe that zinc associated with pigment is unaffected by the preservation method. Indeed, the longevity of zinc-pigment co-localization has even given rise to zinc being used as a biomarker for melanin-related pigments in fossil remains, for example in *Archaeopteryx* feathers [32]. Other zinc levels, however, are affected by the method of preservation, as zinc levels were lower in methacrylate sections for liver but not for kidney sections.

**Figure 5:**
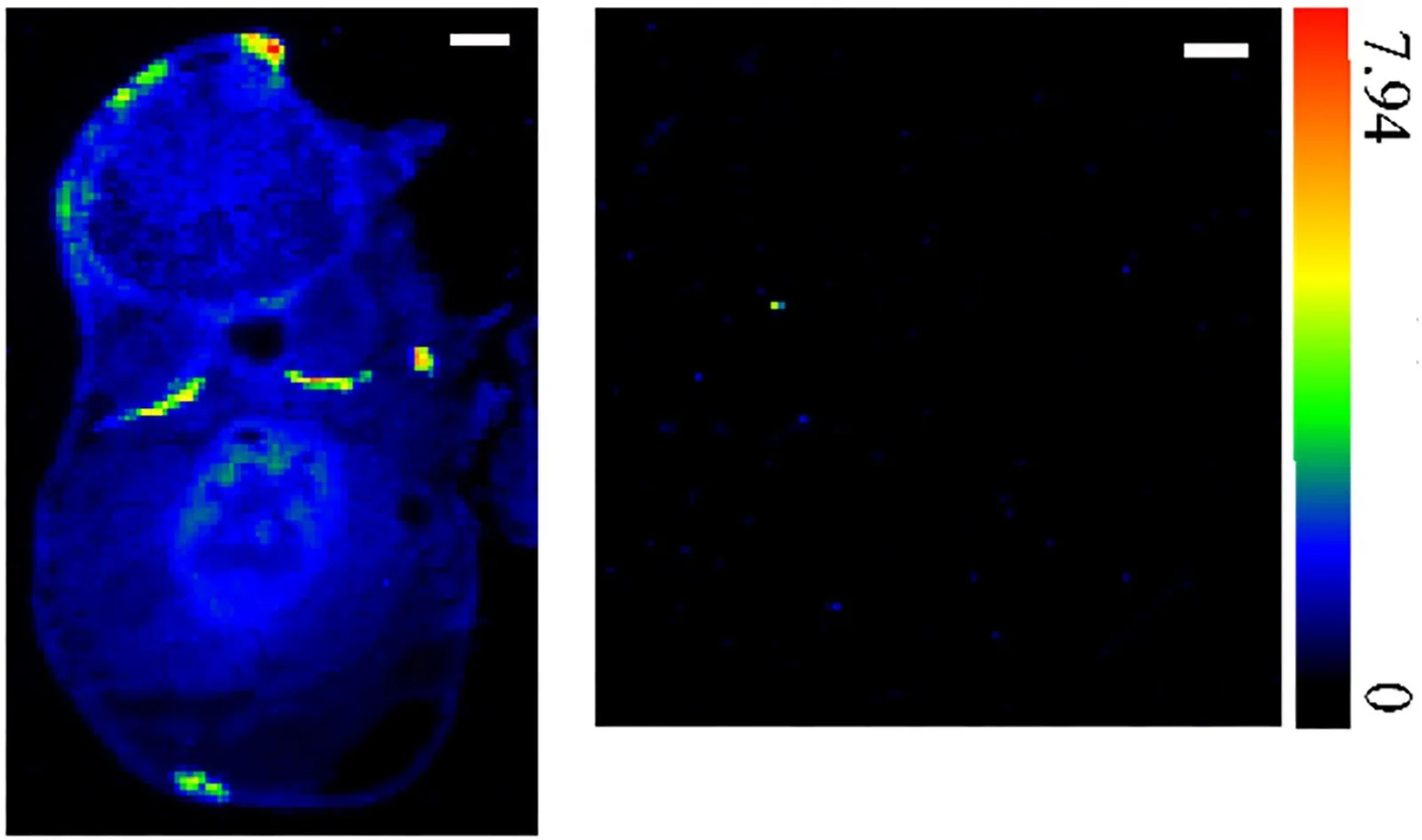
Potassium XFI maps of the same zebrafish trunk sections shown in Fig. 3 (C, D). The abundant potassium observed in the cryosection sample (left) is essentially depleted in the methacrylate sample (right). Scale bar = 50 µm.

All of copper, calcium and potassium are significantly depleted with methacrylate relative to cryopreserved sections. This difference is most marked for potassium with measurements falling between 15% and 1% of the levels in the cryosections (Fig. 4). Figure 5 shows an example of this with the trunk sections showing near-complete potassium depletion with methacrylate preservation.

Methacrylate embedding involves multiple processing steps that may redistribute or leach mobile elements from tissue. Although information on methacrylate effects in XFI is limited, its initial paraformaldehyde fixation step shares similar chemistry with formalin fixation through methylene cross-linking of proteins, suggesting that cautious comparisons are appropriate.

Mercury concentrations and distributions were largely unchanged between cryopreserved and methacrylate-preserved sections (Fig. 3), indicating limited mercury mobility following uptake into tissues. Similar stability of mercury during fixation has been reported previously for formalin-preserved brain samples [33,34], at least in the short term. In contrast, lower concentrations of selenium, sulfur, zinc, calcium, copper, and especially potassium were observed in methacrylate-preserved trunk sections relative to cryopreserved tissue. Potassium loss was particularly striking and is consistent with previous reports of leaching of soluble ions during fixation. Sulfur was significantly reduced in brain tissue, likely reflecting loss of mobile sulfur-containing species such as glutathione, whereas sulfur levels in the eye lens were largely unchanged, consistent with incorporation into less mobile proteinaceous forms. Calcium distributions also differed substantially, with diffuse low-level calcium in cryopreserved sections largely absent following methacrylate embedding.

Zinc showed only modest overall changes, although zinc associated with pigment spots, likely melanophores, appeared sensitive to preservation method. Interestingly, Zn and other trace metals have also been associated with eumelanin residues in fossil tissues, where they may persist as biomarkers of original pigmentation [32].

Overall, methacrylate embedding provided excellent anatomical preservation and reliable Hg measurements but introduced variable redistribution or loss of several biologically important elements. Cryopreservation therefore remains preferable when preservation of native elemental distributions is the primary objective.

## Data Availability

Data supporting the findings of this study are available from the corresponding author upon reasonable request.
